# Manipulating Water Wave Propagation via Gradient Index Media

**DOI:** 10.1038/srep16846

**Published:** 2015-11-25

**Authors:** Zhenyu Wang, Pei Zhang, Xiaofei Nie, Yongqiang Zhang

**Affiliations:** 1 College of Civil Engineering and Architecture, Zhejiang University, Hangzhou 310058, People’s Republic of China

## Abstract

It is challenging to realise the perfect manipulation of water waves within a broad range of frequencies. By extending conformal transformation principles to water waves, their propagation can be controlled via gradually varying water depths, permitting the realisation of a desired refractive index profile for linear water surface waves. Wave bending, directional wave emission and wave focusing are analysed experimentally with accompanying simulations. The results demonstrate desired wave manipulations within a broad range of frequencies, confirming the accuracy and effectiveness of conformal transformation for water waves.

The path of light propagating through a medium is not always linear; it can be regulated by carefully arranging the spatial distribution of the medium’s optical property. This concept has highlighted transformation optics as a powerful mathematical technique with applications in the design of artificial optical devices with versatile functions^1^,^2^,^3^. Intriguing devices such as invisibility cloaks^4^,^5^, illusion generators^6^,^7^ and rotators^8^,^9^ usually require complex, anisotropic distributions of permittivity and permeability, which are extremely challenging as far as experimental realisation is concerned. Although some simplified schemes have been proposed, the fabrications were realised at the expence of important optical functions. On the other hand, the devices designed utilising conformal transformation optics^10^, such as carpet cloaks^11^,^12^, require only isotropic dielectrics with a gradient refractive index (GRIN), which may allow for simpler experimental realisations.

Like electromagnetic waves, water waves are a classic kind of wave and have a long and detailed research history behind them. Many factors, which include wind, water depth, underwater terrain and the tides and currents affecting the water wave process, have been studied in order to understand the basic physics of wave propagation and the interaction between waves and structures by using analytical methods, water wave tank experiments and numerical simulations^13^,^14^,^15^,^16^,^17^,^18^,^19^,^20^,^21^,^22^. Due to the highly destructive force that water waves possess, as well as the enormous potential energy reserves that await efficient harvesting, the manipulation of water waves has recently attracted a great deal of attention. Most manipulation of water waves has, hitherto, been based on the band features of periodic structures, which were mainly inspired by photonic crystals and metamaterials. The corresponding phenomena observed in these structures include water wave blocking^23^,^24^,^25^,^26^,^27^, superlesing effects^28^, self-collimation^29^ and directional radiation^30^,^31^,^32^. In addition, the properties of periodic structures in the long wavelength limit have also been investigated for water wave refraction^33^ and focusing^34^,^35^. Recently, interesting phenomena such as negative effective gravity^36^,^37^, epsilon-near-zero focusing^38^ and zero refractive index^39^ have been reported, suggesting new mechanisms for controlling water waves. Moreover, transformation media, derived from transformation optics, have been proposed to develop water wave devices^40^,^41^ such as invisibility cloaks^42^, invisible rotators^40^ and reflectionless shifters^43^, using the homogenization theory, which models an effective anisotropic fluid character. Generally, most water wave control devices mentioned above lead to periodically abrupt changes of water depths, with energy loss presenting additional concerns. It is always challenging to realise the perfect manipulation of water surface waves within a broad range of frequencies.

As a wave travels from deep to shallow water, its velocity and wavelength decrease, which is somewhat analogous to the propagation of light travelling from a low refractive index medium to a high medium. In fact, the governing equations for linear water surface waves take the same forms as the single polarisation Maxwell’s equations, which are form-invariant. Using conformal transformation principles, the versatile manipulation of water waves can be achieved within a fluidly inhomogeneous and isotropic medium, which may be gradually varied water depths equivalent to GRINs. As modern precision machining allows relatively simple staging of a smooth-bottomed profile, the subsequent implementation of conformal transformation devices for water waves becomes much easier and more efficient.

In this report, the conformal transformation principles are extended to linear water surface waves and used to develop wave-functional devices, generating gradually varied water depths to achieve desired refractive index profiles. Based on simulations and experiments, controlling the propagation of water waves through GRIN devices for bending, directional emission, and focusing are confirmed in a broad range of frequencies with negligible losses. Potential applications of such devices may range from shoreline protection to energy harvesting.

## Results

### Device for bending water waves

**Figure 1:**
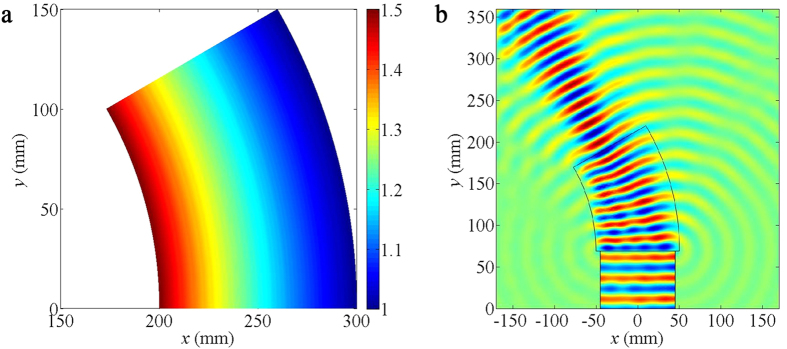
Numerical simulations of wave bending. (**a**) The refractive index distribution of the bending device. (**b**) The simulated spatial distribution of linear liquid surface waves with wavelengths of 25 mm, as they propagate from the bottom edge and traverse the bending device.

The bending device, designed via conformal mapping, has the refractive index distribution^44^:




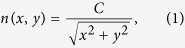




where *C* is a constant that can be used for adjusting height and index ranges within the device. To bend the wave with an angle of π/6, the bending device should be one-twelfth of a circular ring with an inner radius of 200 mm and an outer radius of 300 mm, and *C* should be carefully established at 300, so that the bending device possesses a small index of variation, ranging from 1 to 1.5, as illustrated in Fig. 1(a). Figure 1(b) portrays a simulation of the bending effect for water waves with wavelengths of 25 mm. As incident waves from the bottom edge travel across the device, they are smoothly bent.

**Figure 2:**
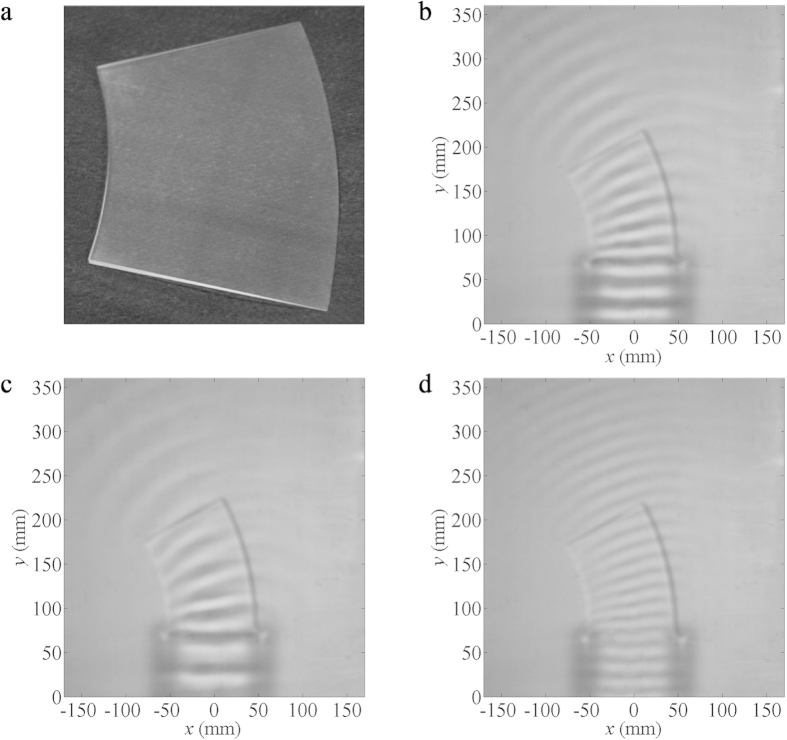
Experimental results of wave bending. (**a**) The photo of the sample bending device. (**b**–**d**) The snapshots of water wave patterns for the bending device at (**b**) 9.12 Hz, (**c**) 6.28 Hz and (**d**) 11.50 Hz, when a linear source is placed about 7 cm away from the bottom edge of the bending device.

Since GRIN can be applied to water waves utilising gradually varied depths, a sample bending device with a sloping surface (as shown in Fig. 2(a)) for smoothly changing water depths, is fabricated and employed to verify theoretical correctness experimentally. Corresponding experimental results are presented in Fig. 2(b–d). Since the water occupies a capillary distribution, the following nonlinear dispersion relation should be considered as providing a suitable explanation:




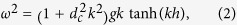




where *d*_*c*_ is the capillary length with *d*_*c*_ = 2.73 mm and *h* is the water depth.

In a water depth of 6 mm, the corresponding frequency for water waves with a wavelength of 25 mm would thus be 9.12 Hz by Eq. (2). A snapshot of wave patterns for the sample bending device at 9.12 Hz is provided in Fig. 2(b). Planar waves are emitted from the upper edges of the bending device, in accordance with the previously simulated result presented in Fig. 1(b). In addition, spatial distributions of water waves at lower and higher frequencies are measured, and the results are shown in Fig. 2(c,d) for 6.28 Hz and 11.50 Hz, respectively. In contrast to ideal situations, wave bending effects remained valid at all operating frequencies in the experiments, apart from small variations of angles, such as the bent angle approximately 26.5° at 11.50 Hz. According to our observations, the bent angle gradually diminishes as the wave frequency exceeds about 11 Hz. This is mainly owing to the fact that the approximation of linear dispersion decreases in accuracy as *k* increases.

### Device for directional emission of water waves

**Figure 3:**
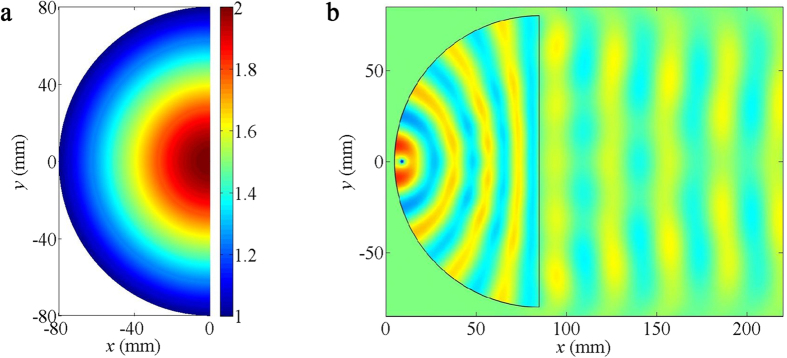
Numerical simulations of directional wave emission. (**a**) The refractive index distribution of the directional emission device. (**b**) The simulated spatial distribution of linear liquid surface waves, when a point source is situated near the vertex of the device and the operating wavelength is 31 mm.

The directional emission device of water waves is derived from Maxwell’s fish-eye lens formula^45^:




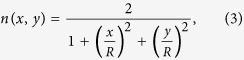




where *R* represents the radius of the device with *R* = 80 mm in our experiment. As illustrated in Fig. 3(a), the refractive index of the device varies from 1 to 2. A point source is placed near the vertex of the device, and the semi-circular boundary of the device is blocked to prevent backward radiation. The simulated result of directional waves emission with wavelengths of 31 mm is presented in Fig. 3(b). Obviously, curved wave fronts gradually flatten as they propagate over the device, and the directional beam appears outside the device.

**Figure 4:**
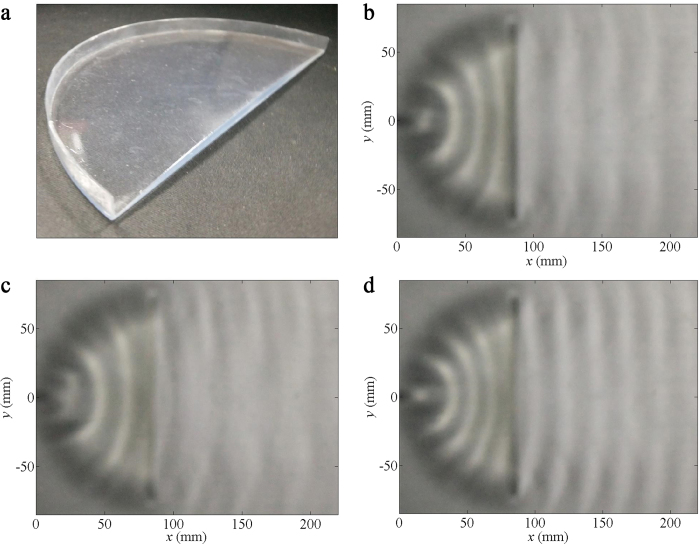
Experimental results of directional wave emission. (**a**) Images of the sample device for directional emission of water waves. (**b**–**d**) Snapshots of water surface wave patterns at (**b**) 7.43 Hz, (**c**) 6.20 Hz and (**d**) 9.90 Hz, when a point source is embedded near the vertex of the device.

A sample device is fabricated in order to realise a directional water wave emission in practice. The photo of the device is presented in Fig. 4(a). Figure 4(b) illustrates the snapshot of wave patterns utilising the device at 7.43 Hz, with liquid wavelengths of 31 mm, as investigated in the previous simulation shown in Fig. 3(b). Similarly, obvious directional beams exiting from the straight boundary of the device are observed, with no variation between the effects of the ideal result (Fig. 3(b)) and those of fabricated samples. The experimental observations shown in Fig. 4(c,d) for 6.20 Hz and 9.90 Hz, respectively, also present nearly flat wave fronts, which provide ample evidence that the directional emission is effective in a broad frequency range. Under our experimental conditions, wave patterns emitted from the device gradually fail to maintain a regular planar pattern when the frequency exceeds about 9 Hz.

### Device for focusing water waves

The focusing of water waves can be seen as an inverse process of wave collimation. It can be realised by using a flat GRIN medium with a refractive index profile of^46^




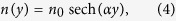




where *α* is a constant defined as




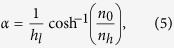




**Figure 5:**
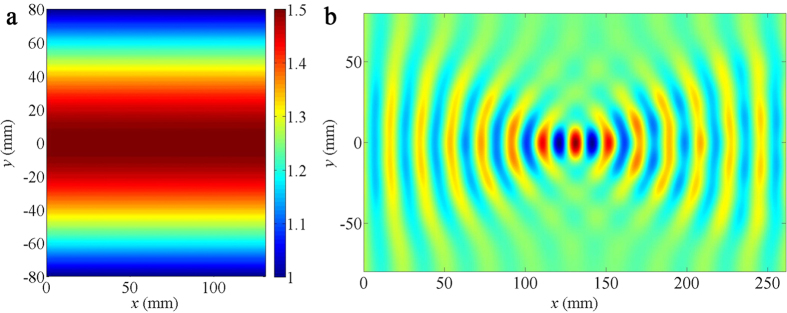
Numerical simulations of wave focusing. (**a**) The refractive index distribution of the device for focusing water waves. (**b**) The simulated spatial distribution of linear liquid surface waves with wavelengths of 28 mm, as they propagate from the left edge and traverse the device with a double focal length (261.14 mm).

*h*_*l*_ is the half-height of the medium, *n*_0_ is the refractive index on the *x*-axis (*y* = 0) and *n*_*h*_ is the refractive index at the medium edges (*y* = ±*h*_*l*_). The device for focusing water waves employed in this study is designed with *h*_*l*_ = 80 mm, *n*_0_ = 1, and *n*_*h*_ = 1.5. The distribution of the refractive index of the device with a focal length of *l*_*f*_ = π/2*а* = 130.57 mm is shown in Fig. 5(a). The simulated results for water waves with wavelengths of 28 mm propagating over the device are presented in Fig. 5(b). Within the device, the plane incident waves parallel to the *y*-axis bend gradually toward the *x*-axis where the refractive index is highest, and converge at a focal point, as shown in Fig. 5(b). After they propagate an additional focal length, focused waves are redirected perpendicularly towards the direction of the propagation.

**Figure 6:**
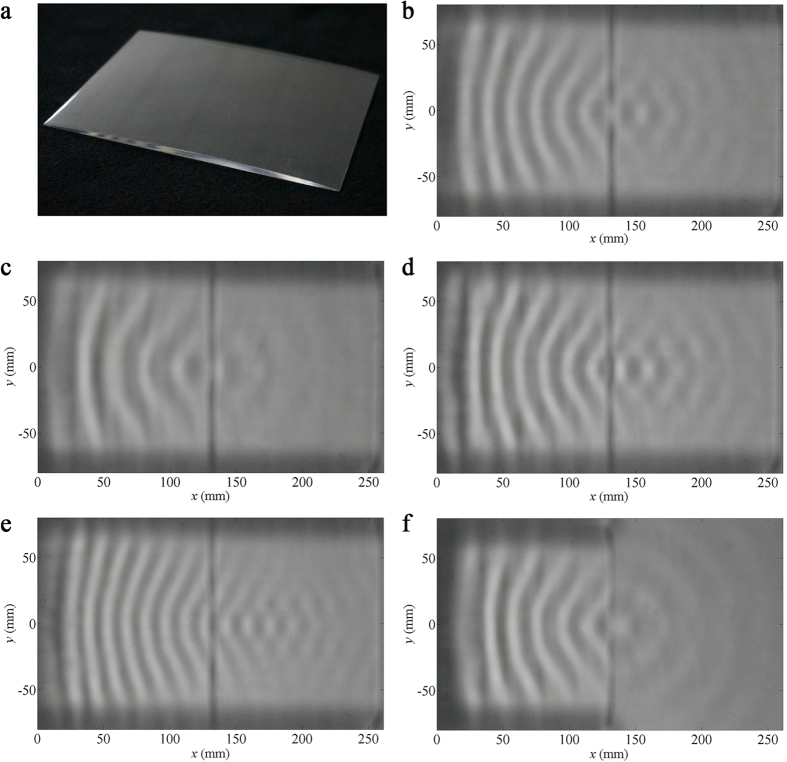
Experimental results of wave focusing. (**a**) Images of the sample device for focusing water waves. (**b**–**e**) Snapshots of water wave patterns at (**b**) 8.18 Hz, (**c**) 6.62 Hz, (**d**) 9.50 Hz and (**e**) 11.50 Hz when the plane waves propagate from the left edge and traverse the device with a length of 261.14 mm. (**f**) Snapshots of water wave patterns under the same conditions as (**b**), except that the device has a focal length of only 130.57 mm.

An experimental verification of the focusing of water waves can also be performed by using the sample device shown in Fig. 6(a). Corresponding experimental observations are illustrated in Fig. 6(b–e), as the plane waves propagate from the left edge and traverse the device with a double focal length of 261.14 mm. The snapshot of water wave patterns presented in Fig. 6(b) is obtained with wavelengths identical to the previous simulation shown in Fig. 5(b). In accordance with the simulated result, the waves bend smoothly and converge at a certain point in the centre of the observed area. The wave patterns shown in Fig. 6(c–e), in which the wave frequencies are 6.62 Hz, 9.50 Hz and 11.50 Hz, respectively, also present the focusing effects; the main difference among them is the practical focal distance. In Fig. 6(b,c), the focal distances observed are almost identical to the theoretical calculation, whereas the focal points in Fig. 6(d,e) are obviously shifted to the right of the ideal position. The reason for this discrepancy will be discussed in the following section. As modelled in the simulation, waves passing the focal position are gradually collimated and revert to planar waves. However, due to the dissipation of wave energy, the wave patterns near the right edge of the device are so unnoticeable that the re-collimated waves cannot be observed clearly. Even so, the collimation of the waves can also be estimated since the wave patterns observed in Fig. 6(b,c) are symmetrical along the middle line (the black line in the middle of the snapshot). Meanwhile, when the water waves traverse the device with only one focal length of 130.57 mm, the wave patterns are also measured at 8.18 Hz as a comparison and presented in Fig. 6(f). Compared to the symmetrical patterns shown in Fig. 6(b), the wave patterns observed in the right half in Fig. 6(f) are noticeably different. There are semicircular wave patterns that, like the waves emitted from a point source, further indicate the effectiveness of the focusing ability of the device.

## Discussion

The experimental results indicate that the desired manipulation of water wave propagation can be efficiently accomplished within a certain frequency range. In our experiments, the water depth *h* ranges from 1.5 mm to 6 mm, with the amplitude of water waves *A* being less than 0.5 mm and wavelength *λ* ranging from 16 mm to 31 mm. We can see that the wavelength is much larger than the water depth, so the water wave can be described in this paper by the shallow water equation. To evaluate the degree of nonlinearity further, we calculate the Ursell numbers according to the formulas^38^ Ur = *Aλ*^2^/*h*^3^. The calculated Ursell numbers are 1.4–35.6  100, which validates the fact that the linear wave theory is applicable.

The effective manipulation frequency ranges for directional wave emission and wave focusing are a bit narrower than those of the bending device. As the design methods and experimental conditions for the devices are nearly identical, such discrepancies are likely to be caused by a linearised approximation of the wave dispersion relation^47^. This approximation affects the practical results in two ways: Firstly, respective refractive index profiles slightly influence the outcomes. The device for directional wave emission, with a larger refractive index variation (from 1 to 2) than that of the bending device (from 1 to 1.5), requires the establishment of greater variations in water depth. As the frequency increases, non-linear effects in the water possess a greater apparent influence on effective water depth, thereby influencing the effective refractive index of the device designed in accordance with the linear dispersion relation. Thus, there are greater differences in the refractive index profile at higher frequencies providing the water depth has a large range of variation; a device with a greater variation of refractive index may operate efficiently in a narrower effective frequency region. Secondly, minor errors accumulate as the wave propagation distance increases. Due to the linearisation approximation mentioned before, there are slight differences between the desired and effective refractive indices of the devices we designed. Athough the differences are nearly negligible at lower frequencies, their accumulation as the waves propagate will inevitably produce minor deviations. The wave focusing device, which has the same variation range of refractive indices as the bending device, cannot focus waves upon a theoretical position when the frequency exceeds 9 Hz. In contrast, the wave bending device still works well within the frequency range of 9–10 Hz, since the smallest distance of the wave propagating over the bending device is 104.7 mm, shorter than that of 130.57 mm in the focusing device.

In our experiment, device sizes and water depths are specified for ease of realisation and observation. In practical coastal engineering applications, the water depth *h* is usually in the order of several metres. In the event of the sizes of the wave bending device and water depth increasing by a factor of 100 (corresponding *h* = 6 m), the equivalent effective frequency should be under approximately 0.907 Hz. Additionally, under such conditions, the capillary length of water (0.00273 m) is so minute that exerted capillary effects may be neglected. A better correlation between experimental and simulated results, as well as a wider effective wavelength range, may be reasonably expected.

The GRIN method can be further explained by comparing it with some of the recent literature written on the subject of water waves. There are a number of publications on the refraction of water waves produced by slowly varying currents and depths^13^,^14^,^15^,^16^. However, these publications are investigations aiming to explain and predict ocean wave phenomena on the basis of the observed data of the sea bed and wind waves. The propagation paths (wave rays) of water waves have been determined by using the geometric optics approach, i.e. ray tracing^13^,^14^. Ray tracing is essentially an approximate solution to wave equations, and its performance will be adversely affected when used in complex media and intricate wave fields, both of which require the application of wave theory. Although water wave bending and focusing have, indeed, been reported previously, the theory and idea of implementation in this paper are different from what has previously been put forward. The requirement of the bending effect for the reflectionless shifter^43^ is an anisotropic water depth distribution deduced from coordinate transformation. The prerequisite for implementing focusing by using epsilon near zero material^38^ is in marked contrast to the water depth deduced by using an analogy between the wave equations of water and electromagneticism. The combination of conformal transformation and GRIN is a powerful tool for designing sophisticated wave manipulation devices. The present wave devices are designed based on isotropic GRIN distributions, which would not encompass abrupt changes of water depth. Seen from the perspective of the effect of the experiment and the flexibility of realisation, the GRIN method put forward in this paper has a number of advantages.

In conclusion, devices for bending and controlling directional emission and the focusing of water waves are experimentally realised by constructing the GRIN medium for the waves in accordance with conformal transformation principles. These wave-functional devices with gradually varying depths produce the desired refractive index profiles for water waves, manipulating their propagation. Experimental observations and numerical simulations demonstrate effective wave control capabilities as well as the broadband property of working frequencies. The propertities of such devices can be used to alter wave directions for applications in shoreline protection and wave energy generation.

## Methods

### Design and fabrication of the sample devices

The equation of linear liquid surface waves employed for mapping is the shallow water wave equation (rigorous when *kh* ≪ 1),




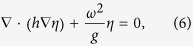




where *h* is the depth of the water, *η* is the vertical displacement of the water surface, *ω* is the angular frequency, and *g* represents gravitational acceleration. The liquid surface waves in the above equation have a linear dispersion relation of 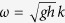, where *k* is the wave number. Based on this equation, the square of the refractive index for the water wave is inversely proportional to the water depths. Accordingly, required water depth distributions may be intuitively derived from the given refractive index profile *n*(*x*, *y*) and external water depth *h*_e_. The height distribution of the wave control device can thus be obtained:




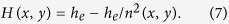




All the sample devices were fabricated by means of the computer numerical control (CNC) technique with transparent polymethyl methacrylate (PMMA), permitting the observation of wave pattern changes within the devices. In order to improve the accuracy and reliability of experimental results, the molding precision for the devices is less than 0.1 mm.

### Numerical simulations

From Eq. (6), it is known that the vertical displacement of a water surface satisfies a two-dimensional Helmholtz equation. The simulated wave patterns can thus be calculated by using the finite-element method. In this study, we utilise PDE interfaces of COMSOL Multiphysics for simulations. The parameters of the simulations, including water depths, device sizes, positions of the sample devices and wave sources, etc., are all in agreement with experimental conditions. All the borders of the simulated region are perfect absorbing boundaries. For the vertical walls above the water surfaces, a no-flow condition is applied to their surfaces.

### Experimental set-up

The experiment is carried out in a horizontally placed vessel covered with 6 mm of water. This vessel consists of a bottom with a size of 700 mm × 400 mm and four slanted sides made of transparent polymethyl methacrylate. All the sides have a length of 120 mm and height of 10 mm, so that the reflection of the water waves can be effectively reduced. The sample devices are placed in the centre of the vessel.

A plane wave generator of 8 cm in length and a point source generator with a diameter of 4 mm are employed, and their amplitude and frequency are tuned precisely utilising a signal generator. Both wave generators can produce stable and easily visualised harmonic water waves within the frequency region of 5 Hz to 15 Hz.

An LED lamp is hung 1.2 m above the vessel so that the light passing through is reflected onto a screen, permitting ready visualisation of projected water wave pattern images. A digital camera was used to record wave patterns reflected on the screen. Experimental results, such as the wave patterns shown in Figs 2, 4 and 6, can be obtained by extracting the corresponding regions from recorded photographs, and the brightness of the wave patterns indirectly reflects the relative strength of the wave amplitude. A similar experimental set-up has been adopted in the previous experiments^28^,^29^,^32^,^35^,^40^. The wave patterns of images taken during such experiments are both straightforward and impressive.

## Additional Information

**How to cite this article**: Wang, Z. *et al.* Manipulating Water Wave Propagation via Gradient Index Media. *Sci. Rep.* **5**, 16846; doi: 10.1038/srep16846 (2015).
